# Barriers and facilitators to enrollment in pediatric clinical trials: an overview of systematic reviews

**DOI:** 10.1186/s13643-024-02698-8

**Published:** 2024-11-20

**Authors:** Veronika Bencheva, Nina-Kristin Mann, Tanja Rombey, Dawid Pieper, Sven Schmiedl

**Affiliations:** 1 Center for Clinical Trials, Faculty of Health, Witten/Herdecke University, Witten, Germany; 2 Chair of Clinical Pharmacology, Department of Medicine, Faculty of Health, Witten/Herdecke University, Witten, Germany; 3 Department of Health Care Management, Technische Universität Berlin, Berlin, Germany; 4 Center for Health Services Research Brandenburg, Brandenburg Medical School Theodor Fontane, Rüdersdorf, Germany; 5 Faculty of Health Sciences Brandenburg, Brandenburg Medical School Theodor Fontane, Institute for Health Services and Health System Research, Rüdersdorf, Germany; 6 Evidence Based Practice in Brandenburg: A JBI Affiliated Group, University of Adelaide, Adelaide, Australia; 7 Philipp Klee-Institute of Clinical Pharmacology, Helios University Hospital Wuppertal, Wuppertal, Germany

**Keywords:** Overview of systematic reviews, Pediatric, Clinical trial, Barriers, Recruitment

## Abstract

**Background:**

Recruiting a sufficient number of patients is often a challenge for conducting clinical trials. Published data reveal that only 10% of eligible patients according to inclusion and exclusion criteria are enrolled in clinical trials. Consequentially, identifying barriers and facilitators may improve enrollment. These factors may differ in the pediatric population, for example, due to the involvement of parents in the decision-making process. We aimed to conduct an overview of systematic reviews to summarize the barriers and facilitators influencing the enrollment of pediatric participants in clinical trials.

**Methods:**

A systematic literature search in PubMed and Epistemonikos of published systematic reviews focusing on barriers and facilitators influencing the enrollment of pediatric patients in clinical trials was conducted. Study selection, data extraction, and quality assessment were performed by two authors independently. The methodological quality was judged using a critical appraisal tool. Finally, data were narratively synthesized.

**Results:**

Of 283 identified systematic reviews, four met the inclusion criteria and were included in the overview. Parents belonging to an ethnic minority or having low socioeconomic status were identified as barriers to enrollment whereas higher parental education and higher age served as facilitators. Additionally, existing expectations, previous treatment experiences and preferences, study duration, type of control group, and the child’s attitude toward study participation could favor or hinder participation. Furthermore, physicians’ opinions of study-related treatments may also influence the enrollment process.

**Conclusion:**

This overview provides a summary of barriers and facilitators to the enrollment of pediatric patients in clinical trials. Taking into account this information may enhance the enrollment of this hard-to-reach population.

**Supplementary Information:**

The online version contains supplementary material available at 10.1186/s13643-024-02698-8.

## Background

Clinical trials (CTs) provide the necessary data to evaluate the efficacy and effectiveness of treatments. To attain robust results, a CT needs to enroll a sufficient number of patients [1, 2], usually determined through a sample size calculation [3]. Sample size calculation is crucial to ensure that a study has sufficient power to detect the desired effect. Failing to achieve the target sample size may lead to premature study termination or the need for extended recruitment, increasing the study costs [3, 4]. Reviews of publicly funded trials indicated that only 31% to 53% reached their original target sample size [4]. A major contributor can be an insufficient participation rate, defined as the proportion of individuals who take part in a study out of the total number of individuals who were eligible to participate. Analyses of completed trials show that participation rates may vary depending on the type of trial and the specific conditions being studied. For studies in oncology, for example, participation rates of 10.9% [2], 3–8% [5], or 2–3% [6] have been reported for different age groups including children among others. A better understanding of the reasons for these low participation rates may help to optimize enrollment and retention in clinical trials.

The inclusion and exclusion criteria define implicitly the number of patients potentially eligible to participate in a CT, but additional factors may also impact the number of patients enrolled in a clinical trial. For example, patient-related (e.g., education) or structural and institutional factors can either restrict or hinder participation, thereby acting as barriers or promote and enable participation, serving as facilitators for patients` enrollment [7]. For adults, there are several published systematic reviews investigating these factors in specific indications such as cancer [2, 5–7] or depression [8]. In addition, an overview summarizing results from systematic reviews has been published [9].

For the pediatric population, these factors may differ and can be expected to be much more complex, e.g., due to the mandatory parental consent needed for a child’s enrollment in a clinical trial. For example, the parents’ preferences play a major role in the decision-making process [10]. Several studies have shown that the decision-making process is more complex and yields different decisions if parents make decisions not for themselves but for their children [11, 12].

Despite some improvement in recent years, there are far too few clinical studies on children, e.g. due to lack of funding and particular ethical issues [13]. An analysis of trials registered in clinicaltrial.gov from 2000 to 2019 showed that only approximately 6% of trials focused exclusively on pediatric participants [14]. Furthermore, only 16.7% of trials registered in the World Health Organization (WHO) portal focused on this population [13]. In contrast to these data, children hold a 27% share of the world population [11]. Compared to trials in adults, pediatric trials were also rather small-scaled and single-sited [15].

Regarding the low number of CTs conducted in children and adolescents, enrollment of pediatric participants is of outstanding importance. Hence, we aimed to conduct an overview of systematic reviews synthesizing the available evidence on barriers and facilitators to the enrollment of pediatric participants.

## Methods

The PRIOR reporting guideline was followed to ensure complete and accurate reporting of this overview of systematic reviews [16]. This review was registered on OSF (https://osf.io/64fb5). We followed an internal protocol that is available from the authors upon request.

### Eligibility criteria

Only systematic reviews were included in the overview. In our context, a systematic review was defined as a review that reports or includes the following:

i) Research question;
ii) Sources that were searched, with a reproducible search strategy (naming of databases, naming of search platforms/engines, search date, and complete search strategy);
iii) Inclusion and exclusion criteria;
iv) Selection (screening) methods;
v) Critically appraises and reports the quality/risk of bias of the included studies;
vi) Information about data analysis and synthesis that allows the reproducibility of the results [17].

For our purpose, eligible systematic reviews had to analyze quantitative or qualitative data on barriers and facilitators to enrollment in pediatric randomized CTs (age < 21 years) or separate this data for patients aged < 21 years according to the American Academy of Pediatrics, where the age limit of pediatrics is defined by the upper age threshold of 21 years [18]. Barriers were defined as factors that can limit or prevent participation in clinical trials. Facilitators were defined as factors that promote and enable enrollment. Hence, SRs focusing solely on retention in CTs were not included in this overview. Due to the huge impact of economic aspects on enrollment in low-income countries, we only focused on high-income countries according to the World Bank’s Classification [19]. Only publications in English or German were included.

### Information sources and search strategy

A search of the literature was conducted in PubMed and Epistemonikos in September 2024 as the combination of both databases is regarded as the most effective approach, according to a study by Goossen et al. 2020 [20]. The search strategy was developed using the search filter for systematic reviews published by Salvador-Oliván et al. 2021 [21] and the criteria described above. The search strategy is outlined in Supplementary material 1.

### Study selection

The relevant publications were selected in two steps: First, titles and abstracts were screened. Then, full-text articles were checked against the inclusion and exclusion criteria. All steps in the selection process were conducted by two reviewers (DP, NM, VB, SS) independently. In case of disagreements between the reviewers, either a third reviewer or the whole author group was consulted.

### Data extraction

Data extraction was not piloted because we did not expect a large number of systematic reviews. One reviewer (NM) extracted relevant data from all selected reviews into an Excel spreadsheet comprising the following data items: first author, publication date, country (defined by the affiliation of the first author), date/time range of the literature search, country or countries of primary studies, medical field, setting, number of included studies and study type (qualitative, quantitative), the aim of the review, barriers, and facilitators to patient participation, study population (e.g., parents, patients, nurses), databases searched, methods for synthesis of results and risk of bias or methodological quality rating tool for included studies. A second reviewer (VB) cross-checked the extracted data. We did not re-assess or extract the results of the risk of bias or methodological quality assessments of the primary studies as we regarded the impact on our outcome of interest—the identification of factors—to be negligible.

### Critical appraisal

The quality of the included reviews was rated using the JBI critical appraisal tool [22]. The tool consists of 11 yes/no questions and does not result in an overall score. Two reviewers (DP and TR) assessed the reviews independently with discussion or arbitration by a third reviewer (SS) in case of any disagreement.

### Synthesis of results

Two reviewers (VB, SS) analyzed the results of the included SRs and a discussion was performed with the other authors (DP, TR, NM). Barriers and facilitators to participation were described narratively in this overview by discussing recurring themes and motives. A deductive procedure considering the identified SRs was chosen to develop the framework for presenting the results of the included SRs in our overview. The themes were summarized in related clusters representing the main points influencing enrollment. We did not perform a formal assessment of overlap as we assumed that the risk of overlap would be low due to the overviews` broad inclusion criteria and the expected diversity of objectives and methods used across the SRs. Due to the narrative nature of our overview, we did not perform any reporting bias or certainty assessment.

## Results

**Fig. 1:**
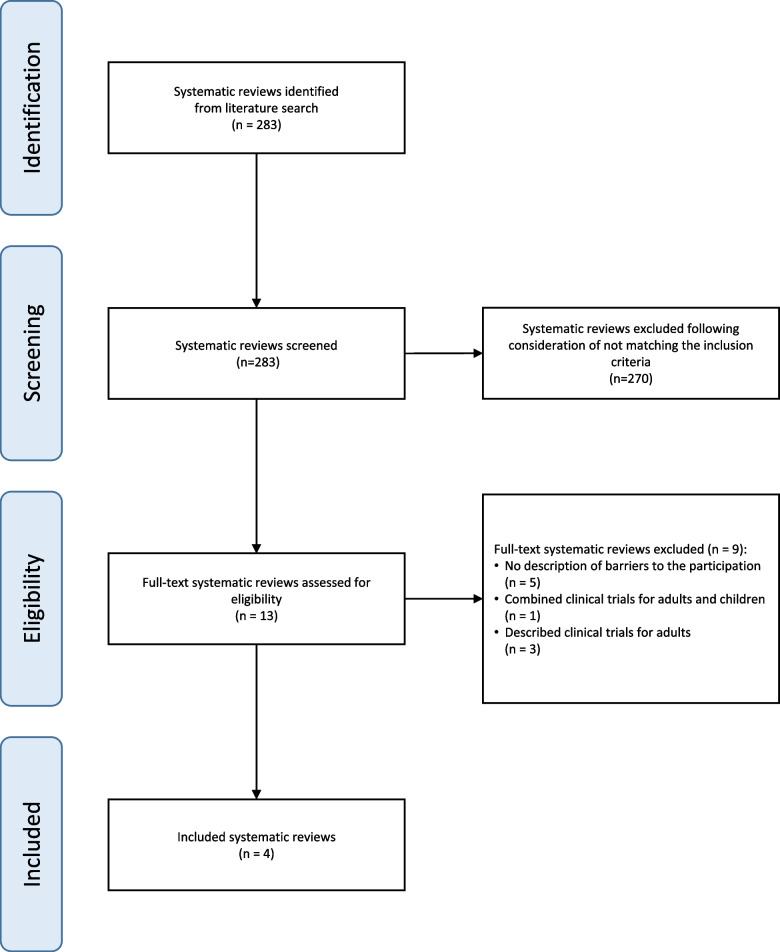
PRISMA Flowchart for the selection of studies

The systematic search retrieved a total of 283 hits. After screening the titles and abstracts, 13 reviews were considered for full-text assessment. After obtaining the full texts we excluded nine further records (Supplementary material 2) that did not match the inclusion criteria. Consequently, four systematic reviews were included in this overview [23–26]. The results of our screening process are shown in the study flow diagram (Fig. 1).

### Description of included reviews

This overview summarizes four SRs, whose number of included studies totaled 162 [23–26]. The four SRs showed heterogeneity in study selection, settings, and the age of the study populations. Robinson et al. (2016) conducted a broad analysis that encompassed all randomized clinical trials (RCTs) involving pediatric populations, differentiating between medical and non-medical studies without further categorization into drug-related or non-drug-related studies [26]. Beasant et al. 2019 similarly focused on pediatric RCTs without making a further categorization of the involved studies [23]. The review of Hanvey et al. 2019 focused specifically on clinical drug trials from Phase I through Phase IV [24]. The review of Le Rouzic et al. 2020 included studies addressing the informed consent and communication between physicians, parents, and children in the context of offering participation in Phase I clinical trials or related to parental decision-making during the palliative phase of oncology care [25].

In addition, the settings of the included studies differ, Beasant et al. (2019) focussed on studies conducted within healthcare services [23], whereas the other three analyzed systematic reviews did not explicitly restrict the settings of the included studies in their search criteria [24–26]. Only one of these reviews provided details on the settings of the included studies, which encompassed home visits, university clinics, hospitals, and educational institutions [26].

The age range in the SRs varied between neonates (birth to 28 days of age) [24], 0–12 years [26], 0–17 years [23], and 0–21 years [25]. Further variability was found for the publication dates and indications covered by the clinical trials included in the SRs [23–26]. Heterogeneity was also found in the evaluated factors influencing the participation in a trial (e.g., treatment preferences, sociodemographic issues, or emotional burden) and will be discussed under outcomes [23–26]. Two of the included reviews focused not only on enrollment but also on retention and discussed these factors separately [23, 26]. Characteristics of the four systematic reviews are given in Table 1. Further details are given in Supplementary material 3.

**Table 1:** Overview of the included sy﻿stematic reviews

Author (s); Title (year); country of the first listed affiliation	Aim of review	Number of included articles (n)	Country/countries included (*n*)	Study type(s) of the included studies	Review population	Factors influencing patient participation
Beasant et al.; “Treatment preference and recruitment to paediatric randomized controlled trials: a systematic review” (2019); United Kingdom [23]	Description of the impact of treatment preferences on recruitment and retention for any indication in pediatric randomized controlled trials	52 papers, 7 of those reported results from multiple studies	17 UK 10 Europe 16 USA and Canada 7 outside of North America and Europe 2 international	Quantitative studies and qualitative sub-studies in randomized controlled trials	Children, and adolescents aged 0–17 were recruited to randomized controlled trials	Preference of treatment as a barrier to patient participation: due to preference of treatment, 2 to 50% of the eligible families refused participation in conventional trials. In comparison, in two pilot/feasibility phase trials, this number ranged from 4 to 70%. If a non-randomised preference arm was included 11 to 55% of the families refused randomisationarms. Child views differed from the parent’s view on three occasions. Retention was barely reported in the included studies
Hanvey et al.; “Trial Characteristics That Affect Parental Consent in Neonatal Drug Trials” (2018); United States [24]	Determination of parental consent rate (median percentage) and identification of trial characteristics which are associated with higher parental consent for any indication in pediatric clinical drug trials	52 studies (reported in 51 papers)	Trials outside Western Europe, Canada, the USA, and Australia were excluded, no further information	Quantitative studies: 4 phase I; 15 phase II; 29 phase III; 4 phase IV	Neonates (birth to 28 days of age)	Study characteristics influencing patient participation: a shorter study duration and active comparator study had a positive impact on parental consent. No significant impact on the parental consent was found regarding “study type (phase I, II, III), gestational age, drug dosing, blood sampling use,randomization, funding source, and maximum length of treatment”. The percentage of parental consent was calculated with a medium of 79% for the included studies
Robinson et al.; “Identifying the participant characteristics that predict recruitment and retention of participants to randomised controlled trials involving children: a systematic review” (2016); United Kingdom [26]	Identification of factors that predict recruitment and retention in RCTs with children in medical or non-medical studies for any indication	28 studies	17 USA 4 Canada 2 Australia 1 Switzerland 1 Germany 1 UK 2 Netherlands	Quantitative RCTs	Children from birth to 12 years	Sociodemographic predictors to patient participation: 66 significant predictors were found of participation for both recruitment and retention. These predictors were divided into parent, child, family, and neighborhood characteristics
Le Rouzic et al.; “Characteristics of parental decision-making for children with advanced cancer who are offered enrolment in early-phase clinical trials: A systematic review “ (2022); France [25]	Description of the parental decision-making process, patient’s place (child or adolescent) in this process, and the optimal physician communication in phase I pediatric cancer trials or palliative phase trials	30 papers	1 Canada 5 France 1 Mexico 1 Switzerland 1 UK 20 USA 1 USA and Australia	3 reviews, 7 expert opinions, 20 research articles (17 qualitative design and 3 mixed methods)	Children, adolescents (0–21 years)	“Characteristics of parental decision-making” influencing patient’s enrolment in clinical trials: the factors influencing the parental decision-making process were described according to three categories: “expectations, cancer experience, and family considerations”. The impact of the physician involved in the decision was evaluated as high. Furthermore, the optimal communication process was described and characterizedby the following categories: “prior assessment, alliance and providing specific information, timing, caring, involving thechild, implementation of palliative care, and need to improve consent care”. The need to involve the child/adolescent in the decision-making process was also ranked high

### The methodological quality of the included reviews

Details of the quality assessment using the JBI critical appraisal tool are presented in Table 2 [22]. None of the included SRs fully met the quality criteria, but each had at least two methodological weaknesses (i.e., questions answered “no”). The item where most SRs had a weakness was the search strategy. Some of the quality criteria were not stated explicitly and therefore rated as unclear. Two questions of the JBI critical appraisal tool were assessed as not applicable to the four included SRs by the two reviewers.

**Table 2:** Critical appraisal of included systematic reviews based on the JBI critical appraisal tool

Nr	Questions	Robinson et al. 2016 [26]	Beasant et al. 2019 [23]	Hanvey et al. 2019 [24]	Le Rouzic et al. 2020 [25]
1.	New question: is the review question clearly and explicitly stated?	Y	Y	Y	Y
2.	Were the inclusion criteria appropriate for the review question?	Y	Y	U	U
3.	Was the search strategy appropriate?	N	Y	N	N
4.	Were the sources and resources used to search for studies adequate?	Y	Y	N	N
5.	Were the criteria for appraising studies appropriate?	U	NA	NA	U
6.	Was critical appraisal conducted by two or more reviewers independently?	U	NA	NA	U
7.	Were there methods to minimize errors in data extraction?	Y	N	U	U
8.	Were the methods used to combine studies appropriate?	N	N	Y	Y
9.	Was the likelihood of publication bias assessed?	NA	NA	NA	NA
10.	Were recommendations for policy and/or practice supported by the reported data?	NA	NA	NA	NA
11.	Were the specific directives for new research appropriate?	Y	Y	Y	Y

*Y *met, *N *not met, *U *unclear, *NA *not applicable

### Synthesis of the main results

The four included SRs focused on different aspects influencing children’s participation in clinical trials (Table 1).

In the SR published by Robinson et al. 2016 [26], 28 studies reporting quantitative results for predictors for participation in RCTs were included. In the context of this SR, participation included both, recruitment (i.e., screening and enrollment) and retention (i.e., remaining). In these 28 studies, 155 participant factors were reported in total with considerable variation of factors tested for significance between the studies. Due to variations in measures used in the studies, the authors abstained from conducting a meta-analysis. The 155 participant factors were classified into characteristics related to parents, child, family, and neighborhood. For recurrent factors across the studies, separate summaries (recruitment versus retention) were depicted in the SR. The factors most frequently reported in recruitment (i.e., enrollment) studies were “parental ethnicity” and “parental education” followed by “parental age” and “child gender”. Interestingly, the proportion of studies with statistically significant results for these common factors differed widely (parental ethnicity: 6 out of 12, parental education: 4 out of 7, parental age: 3 out of 6, child gender: 1 out of 6). In detail, belonging to an ethnic minority was revealed as a barrier in some (mainly non-medical) studies whereas higher parental education and age were identified as potential factors associated with better recruitment. Other recurrent factors, such as neighborhood and family factors were considered in quantitative recruitment studies to a much lesser extent.

The review of Le Rouzic et al. 2020 [25] focused on the parental decision for their children on enrollment in early-phase clinical oncology trials, illustrated the decision process and stated emotional factors affecting this decision. No quantitative analysis was conducted. The analyzed factors were summarized into three clusters: (i) expectations such as hope, altruism, uncertainty, and negative impact, (ii) cancer experiences such as emotional burden, desire to fight, the importance of timing, medical facts and refusal, and (iii) family considerations such as parental attitude, practical issues, child characteristics, relationships, and spirituality. Therapeutic misconception and ethical issues by misinterpreting the study purpose were further topics concerning phase I trials. Trigger factors influencing the parental decision were the physicians’ perception, expectations and attitude. Subsequently, the communication process between physicians and parents through a better therapeutic alliance, empathic attitude and timely delivery of information was summarized as an important factor influencing enrollment. Besides these factors, the place of children or adolescents in this process was described. Their willingness to be involved may depend on their age and expectations. Factors influencing their decision are relationships, expectations, faith, and altruism.

The SR by Hanvey et al. 2019 [24] focused on parental consent rates among different study characteristics for phase I, phase II, and phase III and phase IV trials. Seventy-three percent of trials reported information on the percentage of parents’ consent. The following study characteristics were evaluated: study phase, gestational age, randomization type, drug administration route, drug dosing frequency, blood sampling, active comparator vs. placebo, length of study, funding source and length of treatment. In this SR, a quantitative combined analysis was conducted and statistically significant differences (*p* < 0.05) were found for the active comparator vs. placebo, length of study and blood sample collection. The median percentage of parental consent was 72% for studies including placebo compared to 87% when an active comparator was used. In general, a shorter study duration had a higher percentage of parental consent compared with a longer duration (< 24 h: 100%, 1–7 days: 84%, 8 days–1 month: 93%, > 1 month–6 months: 70%, > 6 months: 53%, unknown study length: 82%). For other factors, e.g., study phase, gestational age, drug administration route, drug dosing frequency, blood, funding source and length of treatment, no statistical differences in participation rates were found. A subanalysis of phase III and phase IV showed a higher parental consent for studies with at least one blood sample collected (83% vs 62%, *p* < 0.05) as well.

The systematic review of Beasant et al. 2019 [23] included qualitative and quantitative studies and ranked the rate of declining participation in clinical trials due to other treatment preferences (e.g., decline randomization, concerns about side effects, decline placebo use) without conducting a meta-analysis. This rate ranged from 2 to 70% overall. In conventional RCTs, 2–50% of the parents declined to participate due to particular treatment preferences. For feasibility studies (i.e., non-conventional RCTs), the rate was up to 70%. In several studies, a non-randomized preference arm was available in addition to a randomized treatment. For these studies, 11 to 55% of the families refused the randomization arm because of a preference for the (non-randomized) treatment. Children’s preferences were underreported and differed on three out of nine occasions. The clinicians’ treatment preferences were also underreported but influenced the parenteral decision. For example, medical staff have doubts regarding randomization and interventional treatments.

**Fig. 2:**
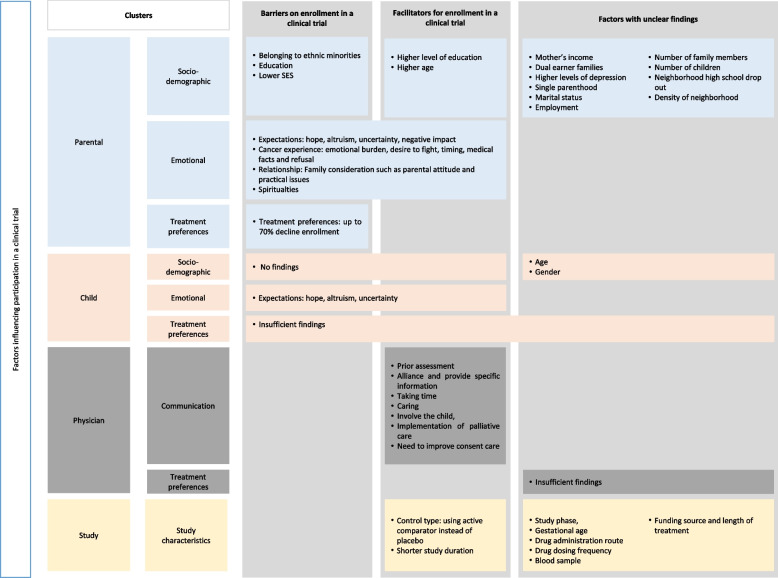
Factors influencing participation in a clinical trial

To sum up the four systematic reviews, we found some quantitative data (including a de-novo calculation) but also several qualitative data. Due to the methodological heterogeneity of the SRs, we tried to synthesize the SR results from a contextual perspective and found some agreements and some discrepancies. All four SRs discussed the parental role as the major factor influencing the decision for enrollment in clinical trials. Similarly, the SRs agreed on the influence of physicians and the importance of the communication process on the decision-making process. The treatment preferences of parents and their children also need to be considered before conducting a clinical trial as they have a significant impact on the decision to participate. In two of the SRs, randomization was identified as a barrier to participation in a clinical trial. The results were thematically summarized in four categories: parental, children, physician, and study-specific factors (Fig. 2). These categories were further sub-classified into sociodemographic factors, emotional factors, and treatment preferences.

## Discussion

To the best knowledge, we provide for the first time an overview of SRs to synthesize the available evidence of published barriers and facilitators regarding the enrollment of pediatric patients in clinical trials. The included four SRs focused on different aspects influencing this process.

Wide ranges of parent, child, family, and neighborhood sociodemographic factors were evaluated to determine their influence on the participation rate. Of these dimensions, the most reported variables were the parent’s sociodemographic factors such as age, income, SES, and education. The SRs included in this overview showed methodological and contextual heterogeneity and insufficient reporting on some factors such as parental depression or marital status. Uniform and detailed reporting of sociodemographic data in clinical trials may contribute to better comparability and evaluation of such parameters.

A further significant factor influencing recruitment is the emotional condition of the family. This may serve as a barrier or a facilitator to participation in a study and may be addressed through better communication.

The high rate of rejection to participate in clinical trials due to treatment preferences may be optimized by including a preference arm or an active comparator group that may be a significant facilitator for parental consent. On the other hand, a placebo-controlled trial may be the best option in indications where a standard treatment still needs to be defined.

Overall, the SRs concluded that few of the available studies reported children’s factors influencing recruitment, their treatment preferences, and expectations [23, 25, 26]. Thus, a detailed analysis of these factors was difficult to conduct and emphasized the need for comprehensive reporting. Le Rouzic et al. 2020 show the possibility of engaging younger adults in the decision-making process [25].

In the SRs, the perceptions and impact of the healthcare professional have been described as a part of the decision-making process. The attitude of the physicians toward the study and their treatment preferences affect recruitment [23]. Personal barriers faced by clinicians were not mentioned in the four SRs summarized in this overview. Further barriers such as lack of reward and recognition, time constraints, and loss of professional autonomy were revealed by the systematic review from Ross et al. 1999 in the adult population [1]. Up to now, the impact of such physician-related factors on participation rates in pediatric studies has not been examined in a well-designed manner. The need for clear communication and assistance throughout the informed consent was pointed out and is supported by the findings of other reviews [1].

Moreover, two of the summarized reviews analyzed factors influencing retention (i.e., remaining in a clinical trial). In the review of Robinson et al. 2016 [26], the influence of sociodemographic factors on retention was described. Similarly to the enrollment, a higher parental education and/or age, and a higher household income were associated with a statistically better retention in some studies, whilst a lower retention was linked to a low SES. Furthermore, inconsistent reporting of dropouts due to treatment preferences was described by Beasant et al. 2019 [23]. The influence of parental characteristics and emotional condition on retention was not reported in the other two studies and requires further investigation [24, 25].

One systematic review identified differences in study designs that may influence parental consent [24]. Statistically significant factors such as the use of active comparators and shorter study duration were identified to favor participation [24]. These findings may be explained by the desire to provide the best treatment and reduce the treatment burden. However, the statistical significance of blood sampling as a facilitator initially appears counterintuitive at first glance. A possible explanation is that these results were derived from Phase III and IV clinical drug trials, where the benefits of a possible treatment may outweigh the inconvenience and pain of injections. Furthermore, some trials included in the publication involved critically ill infants who undergo more intensive therapeutic procedures. Therefore, these findings may not be generalizable to studies involving less severely ill patients.

Additional important aspects, such as inclusion and exclusion criteria, were not covered in the included systematic reviews. These factors could also play a role in poor enrollment rates, as the availability of eligible patients, whose parents can be approached for consent, may be limited.

Overall, the four reviews showed that parental consent depends on several factors: the severity of the disease, the complexity of the study, the perceived benefits, alternative therapeutic options, trust in medical professionals, and the socio-demographic status of the parents. However, how the burden of disease and study complexity interact and affect the decision-making process, was not explored in the analyzed reviews. Future reviews could focus on evaluating the relationship between these factors and their impact on parental consent.

## Limitations

Some limitations apply to this overview’s findings. Although a critical quality assessment of the included SRs was conducted using an established tool, it cannot be excluded that there is a relevant risk of bias in the methods and results of the studies included in the SRs. Furthermore, we considered a combined MeSH and Title search as an appropriate strategy allowing an identification of relevant publications. However, we cannot rule out the possibility that a broader search would have led to more results.

The heterogeneity of studies included in the four SRs considered in our overview may further influence the validity of our findings. In our overview, we focus on clinical trials. However, there is no clear definition of the term “clinical trial”, and this might have an impact on the inclusion of studies in the included systematic reviews. In addition, we did not primarily focus on intervention strategies to enhance pediatric recruitment. However, there will be some overlap of facilitating factors and strategies for improving recruitment. Furthermore, certain factors can serve both as a barrier and as a facilitator, depending on the design, type, and aim of the study.

Clinical trials can be very heterogeneous with some of them covering pharmacological treatments, while others focus on educational interventions which might also hamper their comparability. Furthermore, definitions used for enrollment, recruitment, participation, and retention differ between the four systematic reviews as well as between definitions reported elsewhere. For example, Robinson et al. 2016 [26] defined “recruitment” as “being randomized onto a study and, therefore, the participant had enrolled” leading to a more or less interchangeable usage of “enrollment” and “recruitment”. On the other hand, recruitment and enrollment were also described as distinct concepts with recruitment defined as “the proportion of people who enrolled, out of all people assessed for eligibility” and enrollment defined “as the proportion of people who enrolled out of all people determined to be eligible” [27]. Moreover, two of the SRs focus on specific populations. The review of Harvey et al. 2019 [24] includes neonates and the external validity to other populations seems to be limited. The review of Le Rouzic et al. 2020 [25] focuses on the decision-making process in phase I cancer trials or palliative care, and again, the transferability of results is limited. Furthermore, we focused our search strategy on barriers and facilitators to participation in clinical trials but chose not to assess retention. Hence, results for the factors influencing retention in clinical trials stated in this overview must not be understood as results of a systematic search. In addition, we did not examine the potential multiple inclusion of studies in the SRs evaluated in our overview which may have some impact on our results.

## Implications for research and practice

The heterogeneity of definitions used in the four SRs clearly underlines the need for better standardization of terms used in the context of CTs. Our overview points out that parents’ attitude, background, and therapeutic experiences play a major role in the decision-making process. Acknowledging and overcoming individual barriers could lead to higher enrollment and, in particular, empathic communication and a detailed information process could lead to better acceptance of CTs. In this process, the treating physician’s attitude, time, and communication skills play an important role, so training and specific recommendations for physicians may be beneficial [25, 28]. Furthermore, it was barely reported to what extent the child/adolescent should or need to be involved in the decision-making process. Of course, formal requirements (i.e., Good Clinical Practice guidelines focusing on the need for pediatric consent) have to be considered at least in drug-related studies. But even for non-medical studies, well-conducted analyses examining potential barriers and facilitators from a child’s perspective are urgently needed.

## Conclusion

In conclusion, this overview of SRs summarizes the available evidence on barriers and facilitators to participating in pediatric clinical trials. We summarized the important roles of parental consent, preferences, attitudes, and experiences, as well as the need to involve children in the decision-making process. Additionally, we highlighted how practitioners’ preferences and attitudes along with study characteristics may influence enrollment. A better understanding of them may enhance the recruitment of pediatric patients in clinical trials.

## Supplementary Information

Supplementary Material 1. Barriers to enrollment in pediatric clinical trials-An Overview of systematic review.

Supplementary Material 2. Barriers to enrollment in pediatric clinical trials-An Overview of systematic review.

Supplementary Material 3. Barriers to enrollment in pediatric clinical trials-An Overview of systematic review.

## Data Availability

Template data collection forms are available from the authors upon request. All data collected from the included systematic reviews are reported in the main manuscript.

## Abbreviations

CTs: Clinical trials
RCT: Randomized control trials
SES: Socioeconomic status
SRs: Systematic reviews
