# Supplementary material for: Barriers and facilitators to enrollment in pediatric clinical trials: an overview of systematic reviews
Source: Syst Rev. 2024 Nov 20;13:283. doi: 10.1186/s13643-024-02698-8 (PMC11577732; doi:10.1186/s13643-024-02698-8)
Supplement: Supplementary file 1 — Supplementary Material 1. Barriers to enrollment in pediatric clinical trials-An Overview of systematic review. [file 13643_2024_2698_MOESM1_ESM.pdf]

Barriers and facilitators to enrollment in pediatric clinical trials: An overview of systematic reviews

Supplementary material 1: Search strategy

Search strategy Medline

(("systematic*"[Title] AND "review"[Title]) OR "systematic overview*"[Title] OR "cochrane review*"[Title] OR "systemic review*"[Title] OR "scoping review"[Title] OR "scoping literature review"[Title] OR "mapping review"[Title] OR "umbrella review*"[Title] OR ("review of reviews"[Title] OR "overview of reviews"[Title]) OR "meta-review"[Title] OR ("integrative review"[Title] OR "integrated review"[Title] OR "integrative overview"[Title] OR "meta-synthesis"[Title] OR "metasynthesis"[Title] OR "quantitative review"[Title] OR "quantitative synthesis"[Title] OR "research synthesis"[Title] OR "meta-ethnography"[Title]) OR "systematic literature search"[Title] OR "systematic literature research"[Title] OR "meta-analyses"[Title] OR "metaanalyses"[Title] OR "metaanalysis"[Title] OR "meta-analysis"[Title] OR "meta analytic review"[Title] OR "meta analytical review"[Title] OR "meta-analysis"[Publication Type] OR (("search*"[Title/Abstract] OR "medline"[Title/Abstract] OR "pubmed"[Title/Abstract] OR "embase"[Title/Abstract] OR "Cochrane"[Title/Abstract] OR "scopus"[Title/Abstract] OR "web of science"[Title/Abstract] OR "sources of information"[Title/Abstract] OR "data sources"[Title/Abstract] OR "following databases"[Title/Abstract]) AND ("study selection"[Title/Abstract] OR "selection criteria"[Title/Abstract] OR "eligibility criteria"[Title/Abstract] OR "inclusion criteria"[Title/Abstract] OR "exclusion criteria"[Title/Abstract])) OR "systematic review"[Publication Type]) NOT ("letter"[Publication Type] OR "editorial"[Publication Type] OR "comment"[Publication Type] OR "case reports"[Publication Type] OR "historical article"[Publication Type] OR "report"[Title] OR "protocol"[Title] OR "protocols"[Title] OR "withdrawn"[Title] OR "retraction of publication"[Publication Type] OR "retraction of publication as topic"[MeSH Terms] OR "retracted publication"[Publication Type] OR "reply"[Title] OR "published erratum"[Publication Type])

AND

("infan*"[All Fields] OR "newborn*"[All Fields] OR "new born*"[All Fields] OR "perinat*"[All Fields] OR "neonat*"[All Fields] OR ("infant, newborn"[MeSH Terms] OR ("infant"[All Fields] AND "newborn"[All Fields]) OR "newborn infant"[All Fields] OR "baby"[All Fields] OR "infant"[MeSH Terms] OR "infant"[All Fields]) OR "baby*"[All Fields] OR ("baby s"[All Fields] OR "babys"[All Fields] OR "infant"[MeSH Terms] OR "infant"[All Fields] OR "babies"[All Fields]) OR "toddler*"[All Fields] OR ("minority groups"[MeSH Terms] OR ("minority"[All Fields] AND "groups"[All Fields]) OR "minority groups"[All Fields] OR "minorities"[All Fields] OR "minority"[All Fields] OR "minority s"[All Fields] OR "minors"[MeSH Terms] OR "minors"[All Fields] OR "minor"[All Fields]) OR "minors*"[All Fields] OR ("men"[MeSH Terms] OR "men"[All Fields] OR "boy"[All Fields]) OR ("men"[MeSH Terms] OR "men"[All Fields] OR "boys"[All Fields]) OR ("boyfriend"[All Fields] OR "boyfriend s"[All Fields] OR "boyfriends"[All Fields]) OR "boyhood"[All Fields] OR "girl*"[All Fields] OR "kid"[All Fields] OR "kids"[All Fields] OR ("child"[MeSH Terms] OR "child"[All Fields] OR "children"[All Fields] OR "child s"[All Fields] OR "children s"[All Fields] OR "childrens"[All Fields] OR "childs"[All Fields]) OR "child*"[All Fields] OR "children*"[All Fields] OR "schoolchild*"[All Fields] OR "schoolchild"[All Fields] OR "school child"[Title/Abstract] OR "school child*"[Title/Abstract] OR "adolescen*"[All Fields] OR "juvenil*"[All Fields] OR "youth*"[All Fields] OR "teen*"[All Fields] OR "under age*"[All Fields] OR "pubescen*"[All Fields] OR "pediatrics"[MeSH Terms] OR "pediatric*"[All Fields] OR "paediatric*"[All Fields] OR "peadiatric*"[All Fields] OR "school"[Title/Abstract] OR "school*"[Title/Abstract] OR "prematur*"[All Fields] OR "preterm*"[All Fields])

AND

("trials"[Title] OR "studies"[Title] OR "random*"[Title] OR "RCTs"[Title])

AND

("Parental Consent"[MeSH Terms] OR "informed consent"[Title] OR "Parental Consent"[Title] OR "participat*"[Title] OR "enroll*"[Title] OR "inclusion"[Title] OR "includ*"[Title] OR "recruit*"[Title] OR "involv*"[Title] OR "attend*"[Title] OR "taking part"[Title] OR "barrier*"[Title] OR "recruit*"[Title]) OR “facilitat*”[Title])

Search strategy Epistemonikos

("infan*" OR "newborn*" OR "new born*" OR "perinat*" OR "neonat*" OR "newborn infant" OR "baby" OR "infant" OR "baby*"OR "baby s" OR "babys" OR "infant" OR "infant" OR "babies" OR "toddler*" OR "minority groups" OR "minority groups" OR "minorities" OR "minority" OR "minority s" OR "minors" OR "minors" OR "minor" OR "minors*" OR "men" OR "boy" OR "boyfriend" OR "boyfriend s" OR "boyfriends" OR "boyhood" OR "girl*"OR "kid" OR "kids" OR "child" OR "child" OR "children" OR "child s" OR "children s" OR "childrens" OR "childs" OR "child*"OR "children*" OR "schoolchild*" OR "schoolchild" OR "school child" OR "school child*" OR "adolescen*" OR "juvenil*" OR "youth*" OR "teen*" OR "under age*" OR "pubescen*" OR "pediatrics" OR "pediatric*" OR "paediatric*" OR "peadiatric*"OR "school" OR "school*" OR "prematur*" OR "preterm*")

AND

("Parental Consent" OR "informed consent" OR "Parental Consent" OR "participat*" OR “facilitat*”OR "enroll*" OR "inclusion" OR "includ*" OR "recruit*" OR "involv*" OR "attend*" OR "taking part" OR "barrier*" OR "recruit*")

AND

("trials" OR "studies" OR "random*" OR "RCTs")
