# Supplementary material for: Barriers and facilitators to enrollment in pediatric clinical trials: an overview of systematic reviews
Source: Syst Rev. 2024 Nov 20;13:283. doi: 10.1186/s13643-024-02698-8 (PMC11577732; doi:10.1186/s13643-024-02698-8)
Supplement: Supplementary file 2 — Supplementary Material 2. Barriers to enrollment in pediatric clinical trials-An Overview of systematic review. [file 13643_2024_2698_MOESM2_ESM.pdf]

Barriers and facilitators to enrollment in pediatric clinical trials: An overview of systematic reviews

Supplementary material 2: Excluded systematic reviews

Authors (Year); Country	Study	Reason for excluding
Abdelazeem et al. (2023); USA (1)	Does usage of monetary incentives impact the involvement in surveys? A systematic review and meta-analysis of 46 randomized controlled trials	The review summarized clinical trials for adults and children.
Henley et al. (2023); UK (2)	Assessing Ethnic Minority Representation in Fibromyalgia Clinical Trials: A Systematic Review of Recruitment Demographics	The review did not describe barriers and facilitators to the participation.
Kilicel et al. (2023); CH (3)	Participant Recruitment Issues in Child and Adolescent Psychiatry Clinical Trials with a Focus on Prevention Programs: A Meta-Analytic Review of the Literature	The review did not describe barriers and facilitators to the participation.
Meskell et al. (2024); IE (4)	Factors that impact on recruitment to vaccine trials in the context of a pandemic or epidemic: a qualitative evidence synthesis	The review describes clinical trials for adults.
Mire et al. (2024); USA (5)	A Systematic Review of Recruiting and Retaining Sociodemographically Diverse Families in Neurodevelopmental Research Studies	The review did not describe barriers and facilitators to the participation.
St-Louis et al. (2018); USA (6)	Enrollment and reporting practices in pediatric general surgical randomized clinical trials: A systematic review and observational analysis	The review did not describe barriers and facilitators to the participation.
Tournoux et al. (2006); USA (7)	Factors influencing inclusion of patients with malignancies in clinical trials	The review summarized clinical trials for adults and children.
Wali et al. (2024); AU (8)	A systematic review of recruitment and retention of ethnic minorities and migrants	The review summarized clinical trials for adults and children.

	in obesity prevention randomised controlled trials.	
Zanfardino et al. (2022); CAN (9)	Black Americans' willingness to participate in pediatric sickle cell clinical trials: A retrospective, systematic review.	The review did not describe barriers and facilitators to the participation.

References:

1. Abdelazeem B, Hamdallah A, Rizk MA, Abbas KS, El-Shahat NA, Manasrah N, et al. Does usage of monetary incentive impact the involvement in surveys? A systematic review and meta-analysis of 46 randomized controlled trials. PLoS One. 2023;18(1):e0279128.
2. Henley P, Martins T, Zamani R. Assessing Ethnic Minority Representation in Fibromyalgia Clinical Trials: A Systematic Review of Recruitment Demographics. Int J Environ Res Public Health. 2023;20(24).
3. Kilicel D, De Crescenzo F, Pontrelli G, Armando M. Participant Recruitment Issues in Child and Adolescent Psychiatry Clinical Trials with a Focus on Prevention Programs: A Meta-Analytic Review of the Literature. J Clin Med. 2023;12(6).
4. Meskell P, Biesty LM, Dowling M, Roche K, Meehan E, Glenton C, et al. Factors that impact on recruitment to vaccine trials in the context of a pandemic or epidemic: a qualitative evidence synthesis. Cochrane Database Syst Rev. 2023;9(9):Mr000065.
5. Mire SS, Truong DM, Sakyi GJ, Ayala-Brittain ML, Boykin JD, Stewart CM, et al. A Systematic Review of Recruiting and Retaining Sociodemographically Diverse Families in Neurodevelopmental Research Studies. J Autism Dev Disord. 2024;54(6):2307-21.
6. St-Louis E, Oosenbrug M, Landry T, Baird R. Enrollment and reporting practices in pediatric general surgical randomized clinical trials: A systematic review and observational analysis. J Pediatr Surg. 2018;53(5):879-84.
7. Tournoux C, Katsahian S, Chevret S, Levy V. Factors influencing inclusion of patients with malignancies in clinical trials. Cancer. 2006;106(2):258-70.
8. Wali N, Huda MN, Gill T, Green J, Renzaho AMN. A systematic review of recruitment and retention of ethnic minorities and migrants in obesity prevention randomised controlled trials. Int J Obes (Lond). 2024;48(8):1065-79.
9. Zanfardino S, Mazziotto V, Bodas P. Black Americans' willingness to participate in pediatric sickle cell clinical trials: A retrospective, systematic review. Pediatr Blood Cancer. 2022;69(5):e29580.
