# Supplementary material for: Barriers and facilitators to enrollment in pediatric clinical trials: an overview of systematic reviews
Source: Syst Rev. 2024 Nov 20;13:283. doi: 10.1186/s13643-024-02698-8 (PMC11577732; doi:10.1186/s13643-024-02698-8)
Supplement: Supplementary file 3 — Supplementary Material 3. Barriers to enrollment in pediatric clinical trials-An Overview of systematic review. [file 13643_2024_2698_MOESM3_ESM.pdf]

Barriers and facilitators to enrollment in pediatric clinical trials: An overview of systematic reviews

Supplementary material 3: Additional information for included systematic reviews

Author (s) (Year); Country	Title	Included languages	Date (range) of literature search	Databases searched	Methods for synthesis of results	Tool for rating of study quality	Mentioned population(s)
Beasant et al. (2019); UK (3)	"Treatment preference and recruitment to pediatric RCTs: A systematic review"	No language restrictions (cave: only mentioned in PROSPERO but not in the paper)	1950-2014	MEDLINE, CINAHL, EMBASE, COCHRANE	Relevant numeric data and/or descriptive reports of treatment preferences were extracted into Excel	CASP qualitative research check-list<br>Cochrane Collaboration's tool for assessing risk of bias (cave: only mentioned in PROSPERO but not in the paper)	Young women, infants, families, parents, staff, children, mothers, patients, participants, parents (and adults), orthodontists, practitioners, adolescents, study coordinators, young children
Hanvey et al. (2018); US (4)	"Trial Characteristics That Affect Parental Consent in Neonatal Drug Trials"		7th June 2009 - 5th June 2014	MEDLINE, EMBASE	The following study characteristics were extracted: phase type, gestational age, randomization type, drug administration route, drug dosing frequency, blood sampling, control type, length of study,	not reported	Infants, parents

						funding source, and length of treatment. Chi-square, Fisher's exact, one-way analysis of variance or Kruskal–Wallis tests were used for the analysis.		
Robinson et al. (2016); UK (5)	"Identifying the participant characteristics that predict recruitment and retention of participants to randomised controlled trials involving children: a systematic review"		English	1946 - October week 3 2012 (MEDLINE), 1806 - October week 3 2012 (PsychINFO), no date restrictions (CINAHL, Cochrane)	MEDLINE, PsychINFO, CINAHL, Cochrane Library	Meta-analysis could not be conducted due to the heterogeneity in scales and measures. The most frequently reported variables were reported giving the significance for each study.	Development of quality assessment tool specifically for this review using/adapting two pre-existing checklists	Babies, infants, families, children, parents, female caregivers, 1st time mothers, mothers, pregnant women with or fathers with asthma, parents and caregivers, low income families, divorced mothers, (children in a specific age range)
Le Rouzic et al. (2022); FRA (6)	"Characteristics of parental decision-making for children with	-	French or English	1st January 2000 - 16th June 2019	PubMed, Web of Science, Cairn, Psychinfo, EM Premium	Data were manually coded using a combination of deductive (derived	Critical appraisal skills program's 10 questions for qualitative and systematic research, CASP	Pediatric hematologist and oncologists, parents, adolescents, physicians, children, patients, mother,

advanced cancer who are offered enrolment in early-phase clinical trials: A systematic review “		from published literature) and inductive (emerging from data) coding clusters. Two authors discussed the clusters and took the final decision. Specific themes and subthemes were developed for recurring patterns of interest.		health care professionals, informed consent conferences, bereaved parents, primary caregivers, psychologists, ethicists

References:

3. Beasant L, Brigden A, Parslow RM, Apperley H, Keep T, Northam A, et al. Treatment preference and recruitment to pediatric RCTs: A systematic review. Contemp Clin Trials Commun. 2019;14:100335.
4. Hanvey IB, Aliaga S, Laughon MM, Testoni D, Smith PB, Bauserman M. Trial Characteristics That Affect Parental Consent in Neonatal Drug Trials. Am J Perinatol. 2019;36(7):759-64.
5. Robinson L, Adair P, Coffey M, Harris R, Burnside G. Identifying the participant characteristics that predict recruitment and retention of participants to randomised controlled trials involving children: a systematic review. Trials. 2016;17(1):294.
6. Le Rouzic MA, Claudot F. Characteristics of parental decision-making for children with advanced cancer who are offered enrollment in early-phase clinical trials: A systematic review. Pediatr Hematol Oncol. 2020;37(6):500-29.
